# Effects of Substrate Addition on Soil Respiratory Carbon Release Under Long-Term Warming and Clipping in a Tallgrass Prairie

**DOI:** 10.1371/journal.pone.0114203

**Published:** 2014-12-09

**Authors:** Xiaohong Jia, Xuhui Zhou, Yiqi Luo, Kai Xue, Xian Xue, Xia Xu, Yuanhe Yang, Liyou Wu, Jizhong Zhou

**Affiliations:** 1 Institute of Desertification Studies, Chinese Academy of Forestry, Beijing 100091, China; 2 Shapotou Desert Research and Experimental Station, Cold and Arid Regions Environmental and Engineering Research Institute, Chinese Academy of Sciences, Lanzhou 730000, China; 3 Department of Microbiology and Plant Biology, University of Oklahoma, Norman, Oklahoma 73019, United States of America; 4 Coastal Ecosystems Research Station of Yangtze River Estuary, Ministry of Education Key Laboratory for Biodiversity Science and Ecological Engineering, The Institute of Biodiversity Science, Fudan University, Shanghai 200433, China; University of Maryland, United States of America

## Abstract

Regulatory mechanisms of soil respiratory carbon (C) release induced by substrates (i.e., plant derived substrates) are critical for predicting ecosystem responses to climate change, but the mechanisms are not well understood. In this study, we sampled soils from a long-term field manipulative experiment and conducted a laboratory incubation to explore the role of substrate supply in regulating the differences in soil C release among the experimental treatments, including control, warming, clipping, and warming plus clipping. Three types of substrates (glucose, C_3_ and C_4_ plant materials) were added with an amount equal to 1% of soil dry weight under the four treatments. We found that the addition of all three substrates significantly stimulated soil respiratory C release in all four warming and clipping treatments. In soils without substrate addition, warming significantly stimulated soil C release but clipping decreased it. However, additions of glucose and C_3_ plant materials (C_3_ addition) offset the warming effects, whereas C_4_ addition still showed the warming-induced stimulation of soil C release. Our results suggest that long-term warming may inhibit microbial capacity for decomposition of C_3_ litter but may enhance it for decomposition of C_4_ litter. Such warming-induced adaptation of microbial communities may weaken the positive C-cycle feedback to warming due to increased proportion of C_4_ species in plant community and decreased litter quality. In contrast, clipping may weaken microbial capacity for warming-induced decomposition of C_4_ litter but may enhance it for C_3_ litter. Warming- and clipping-induced shifts in microbial metabolic capacity may be strongly associated with changes in plant species composition and could substantially influence soil C dynamics in response to global change.

## Introduction

As a result of anthropogenic buildup of carbon dioxide (CO_2_) and other greenhouse gases in the atmosphere, the global mean surface temperature has increased by about 0.78°C over the past century and is predicted to increase by another 1.0–3.7°C by the end of this century [1]. The projected warming is likely to stimulate soil respiratory CO_2_ release due to the enhanced decomposition of soil organic matter (SOM), leading to a weakened terrestrial carbon (C) sink and a positive feedback to climate warming [2], [3], [4]. The majority of global models assume that SOM decomposition rates increase with temperature, which stimulates respiratory C release and/or decreases soil C residence time [4], [5], [6]. Nevertheless, the response of soil C release to rising temperature may be also regulated by many other factors, particularly substrate availability and microbial community [7], [8], [9], [10], [11].

Changes in soil C storage are the result of the net balance between inputs from plant production and outputs from decomposition [12], [13]. Warming-induced changes in plant growth and community structure can considerably influence the quality and quantity of substrates [14], [15], which in turn regulates the responses of soil respiratory C release to rising temperature [7], [8], [16]. Meta-analyses have revealed that experimental warming has different effects on plant growth among vegetation types [17]. The different warming responses of plant growth at species level could contribute to the reported diverse trends of soil C release to experimental warming in recent years, including increases [18], [19], [20], decreases [21], [22], [23], [24], and no changes [25], [26]. Thus, it is critical to study other factors regulating the magnitude and direction of the responses of soil C release to warming, such as the diverse responses of soil C pools with different substrate compositions to temperature [23], [27], [28], [29], [30], [31].

Soil C decomposition is not only mediated by substrate quality and quantity, but also by microbial community structure. For example, fungi tend to be dominant over bacteria in the decomposition of recalcitrant organic matter [32]. Due to the higher C use efficiency of fungi than bacteria, any shift in microbial composition could induce changes in soil C storage [33], [34]. Under warming, soil microbial community may have adapted to previous recalcitrant C [9], [35], which cannot be ruled out as a mechanism for explaining the response of soil respiratory C release to warming. The shifts in microbial composition may thus potentially impact responses of soil respiration rates to a warmer climate, which may cause a high uncertainty in prediction of warming-induced responses of soil respiratory C release. Understanding the regulatory mechanisms of soil C release by substrates and the corresponding changes in microbial metabolic capacity is therefore critical for accurately predicting long-term ecosystem C cycling in the future climate. However, since substrate availability is often associated with changes in microbial communities, their respective effects on soil C release are difficult to be differentiated. Thus, the respective effects of substrate and microbial communities on responses of soil respiratory C release under warming are still unclear. However, additional input of different substrates may provide a unique opportunity to indirectly determine the effects of microbial community on soil C release.

Warming has been reported to increase net primary production (NPP) by up to 26% and shift plant community towards more C_4_ species with low-quality litter at most sites [16], [36], [37]. A long-term warming and clipping experiment was initiated on 21 November 1999 in a tallgrass prairie in the US Great Plains in Central Oklahoma [23]. Besides warming-induced shifts in microbial community towards fungal dominance [38], the result of GeoChip analysis (functional genes array) also demonstrated that the abundances of genes involved in labile C decomposition have generally been stimulated by experimental warming [39]. We thus hypothesized that long-term warming-induced shifts towards a more C_4_-grass dominated plant community [16], [36] and the presence of more fungi [38] would decrease soil C release, inducing a negative feedback between warming and C cycles.

## Materials and Methods

### Ethics statement

The study was carried out on a private land, which was donated to the University of Oklahoma for research by the owner, Dr. Edwin Kessler. No specific permission was thus required to access this land for any locations or activities. The field studies did not involve any endangered or protected species.

### Site description

The experimental site was located at the Great Plain Apiaries (34°58′54"N, 97°31′14"W), 40 km from the Norman campus of the University of Oklahoma. The annual mean temperature was 16.3°C, with monthly mean temperature of 3.1°C in January and 28.0°C in July. The annual mean precipitation was 915 mm. The soil was part of the Nash-Lucien complex (sand: 32%, silt: 60%, clay: 8%), which was characterized as having a low permeability rates, high available water capacity, and a deep and moderate penetrable root zone [40]. The grassland was dominated by three C_4_ grasses: *Schizachyrium scoparium*, *Sorghastrum nutans*, and *Eragrostis curvula*, and two C_3_ forbs: *Ambrosia psilostachyia* and *Xanthocephalum texanum*.

### Experimental treatments

The field experiment used a paired factorial design with warming as the main factor nested by the clipping factor (i.e., aboveground biomass clipping). Each treatment had six replicates (i.e., six pairs). Each pair had two 2 m×2 m plots. In each pair, one plot has been warmed continuously by infrared heaters since 21 November 1999 and the other has remained unwarmed as the control. In each warming plot, a single 165 cm×15 cm infrared heater with a radiation output of 100 W m^−2^ was suspended at a height of 1.5 m above the ground. The radiation output and heater height were sufficient to increase soil temperature by about 2°C, which is within the range of the 1.0–3.7°C increase as predicted by the International Panel on Climate Change by the end of this century [1]. A ‘dummy’ heater of the same shape and size as the infrared heater was suspended 1.5 m high in order to simulate the shading effects of the heater. In order to avoid heating effects in the control plot, the distance between the control and warming plots of each pair was approximately 5 m. Each plot was divided into four 1 m×1 m subplots. The plants in two diagonal subplots were clipped at a height of 10 cm above ground yearly, usually in August. The other two were the unclipped controls. The four field treatments in the experiment were unclipped control (UC), unclipped and warmed (UW), clipped control (CC), and clipped and warmed (CW). Further details of the study have been described in references [16] and [23]. The characteristics of soil were showed in Table 1. Soil temperatures were measured using a thermocouple probe (LI-COR 6000-09TC, LI-COR, Lincoln, NE, USA) before we took soil samples in 2009. Volumetric soil water content (% V) was measured using manual Time Domain Reflectometry equipment (TDR 200, Spectrum Technologies, Inc, IL, USA). The rest of the data in Table 1 have been adapted from the reference [37].

**Table 1:** Mean soil moisture, soil temperature, NO_3_ N, NH_4_ N, total nitrogen, and total carbon before incubation under four treatments in May 2009.

	Unclipped control	Unclipped and warmed	Clipped control	Clipped and warmed
Soil moisture (%Vol)	26.23±8.24	24.09±7.88	26.33±6.55	22.99±6.37
Soil temperature(°C)	22.81±1.73	23.04±1.05	23.62±0.85	24.52±1.53
NO_3_-N (mg kg^−1^ dry soil)	5.40±4.80	4.40±2.33	5.50±6.21	4.40±3.28
NH_4_-N (mg kg^−1^ dry soil)	7.90±2.25	9.00±6.90	7.20±3.01	9.40±7.58
Total nitrogen (%)	0.20±0.04	0.10±0.03	0.10±0.05	0.10±0.03
Total carbon (%)	2.70±0.71	2.50±0.87	2.30±1.22	2.30±0.57

### Substrate materials

In June 2009, soil samples were collected from the topsoil (0–15 cm) in the field after excluding surface litter. To decrease the sample size, only four of six pair plots were selected. Two soil cores (2 cm in diameter) were taken from each subplot. In each paired plot, soil cores from the same treatment were mixed to get one composite sample and then passed through a 2-mm mesh. Any visible plant materials were manually removed from the sieved soil. The processed samples were stored at 4°C for one night before subsequent procedures.

The plant materials were collected during the clipping operation in November 2008 from native C_4_ species (*Sorghastrum nutans*) and C_3_ species (*Ambrosia psilostachyia, Aster ericoides*, and *Dichanthelium oligosnathes*). The C_3_ and C_4_ materials from different treatments were mixed, oven-dried at 65°C for 48 hours and then ground. The average C:N ratios for C_3_ and C_4_ materials were 26.20 and 51.46, respectively.

### Laboratory incubation

Four samples from each treatment were used for laboratory incubation (64 samples in total). Quadruplicate 50-g soil samples from each treatment in the same paired plots were placed individually into 250-mL Pyrex bottle and three different supplementary substrates were added individually: glucose, C_3_ material, or C_4_ material, as well as no addition. The amount of supplementary substrates was 1% of dry soil (i.e., 1 g substrate per 100 g dry soil). All bottles were incubated at constant temperature (25°C) and soil moisture (60% water-holding capacity), the optimal conditions maximizing mineralization [41]. The bottles were linked with a programmable automatic incubation system (Micro- Oxymax system, Columbus Instruments, OH, USA). Since both CO_2_ production and oxygen (O_2_) consumption by soil microbes are a function of O_2_ content and the CO_2_ sensors had a problem in the Micro- Oxymax respirometer system, O_2_ consumed in the process of respiration was used to represent respiratory C release from the soil [42]. Respiration in the soil was thus measured through periodic analysis of O_2_ consumption by an electrochemical oxygen sensor configured in the Micro-Oxymax respirometer every 12 hours. Since headspace gas samples would be pumped out and pass a dryer before measurement by the sensors, a 50-mL Pyrex bottle with distilled water was connected to each bottle containing the soil and substrate samples to humidify the returning gas samples. All soils were pre-incubated for 4 days. Incubation of the samples lasted 6 weeks under dark condition.

### Data analysis

The warming-induced change in soil respiratory C release (ΔW) was defined to be the difference between the warmed and unwarmed treatments under either unclipping (UW vs. UC) or clipping (CW vs. CC), and similarly for the clipping-induced change in soil respiratory C release (ΔC) between clipped and unclipped treatments. The percent of soil respiratory C release relative to the control was ΔW ×100/soil C release in the unwarmed treatment or ΔC ×100/soil C release in the unclipped treatment. The apparent loss of added organic C was estimated by subtracting the total CO_2_ C released in a given treatment from that measured in the control [43]. Relative losses of added C were calculated with the following equation [43]: Loss of added C (C_3_, C_4_ or glucose) was calculated by the apparent loss of added organic carbon×100/total C input. The significant effects of four field treatments on rates of CO_2_ efflux in each addition type over the whole experiment were assessed by repeated measures ANOVA. Owing to the paired design of experiment, we used a paired sample *t*-test to examine the statistical significance of warming, clipping, and their interaction on soil respiratory C release as well as substrate addition. The least significance difference value (5%) was calculated to allow comparison of individual means.

## Results

**Figure 1:**
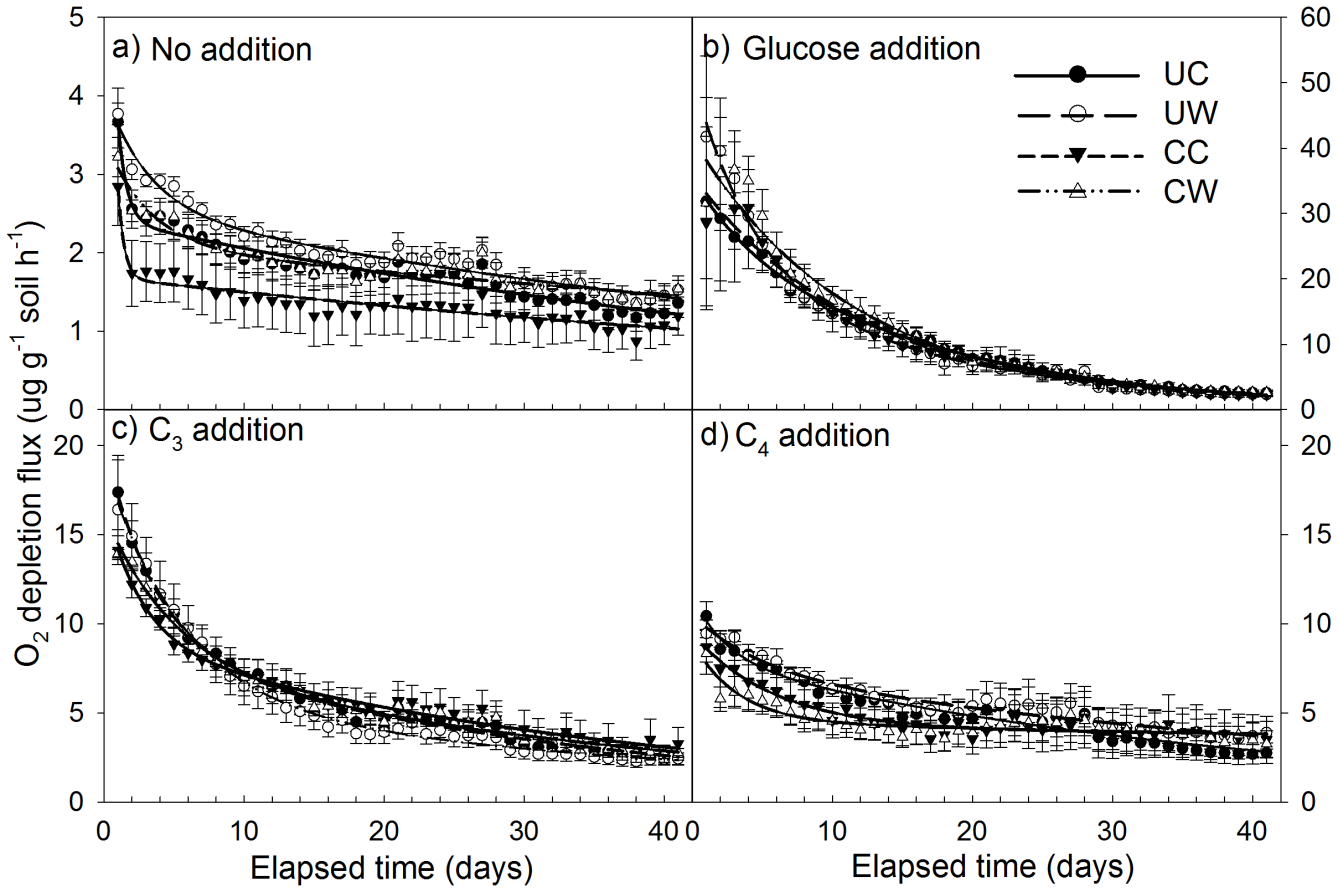
Soil respiratory carbon (C) release represented by O_2_ depletion flux without additional substrate (a) and with the addition of glucose (b), C_3_ (c), and C_4_ (d) substrate under the four treatments: UC: unclipped control; UW: unclipped and warmed; CC: clipped control; CW: clipped and warmed.

**Figure 2:**
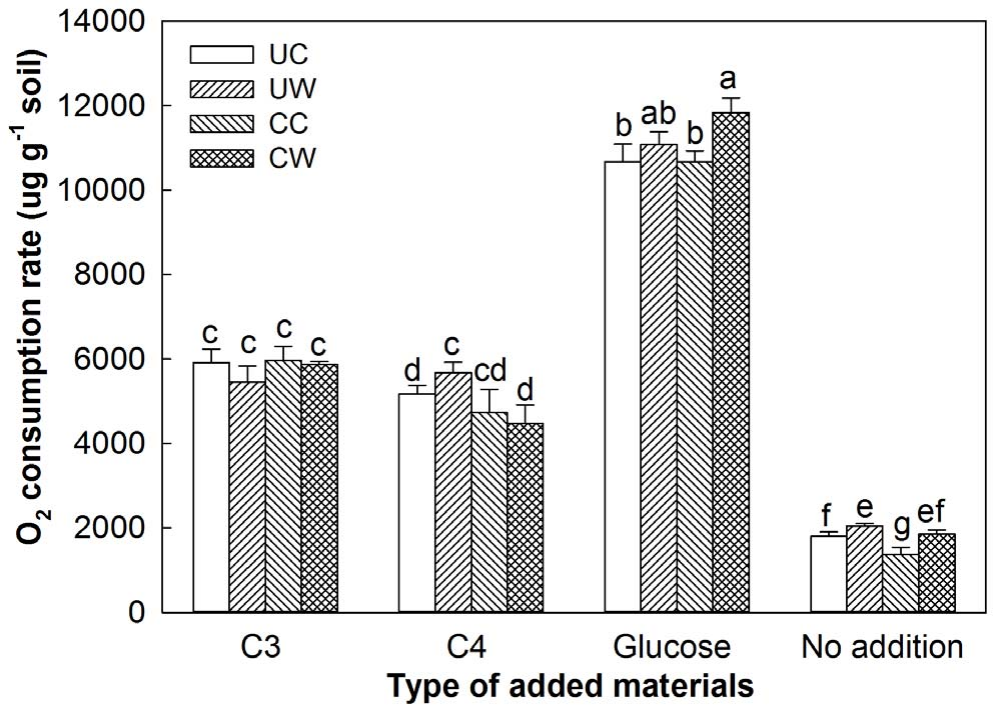
Total soil respiratory C release (i.e., O_2_ consumption rate) with the addition of C_3_ plant material, C_4_ plant material, or glucose or without additional substrate under four treatments during the incubation period. Vertical bars and their error bars represent means and standard errors (*n* = 4). UC: unclipped control; UW: unclipped and warmed; CC: clipped control; CW: clipped and warmed. The different letters indicate statistical significance.

**Figure 3:**
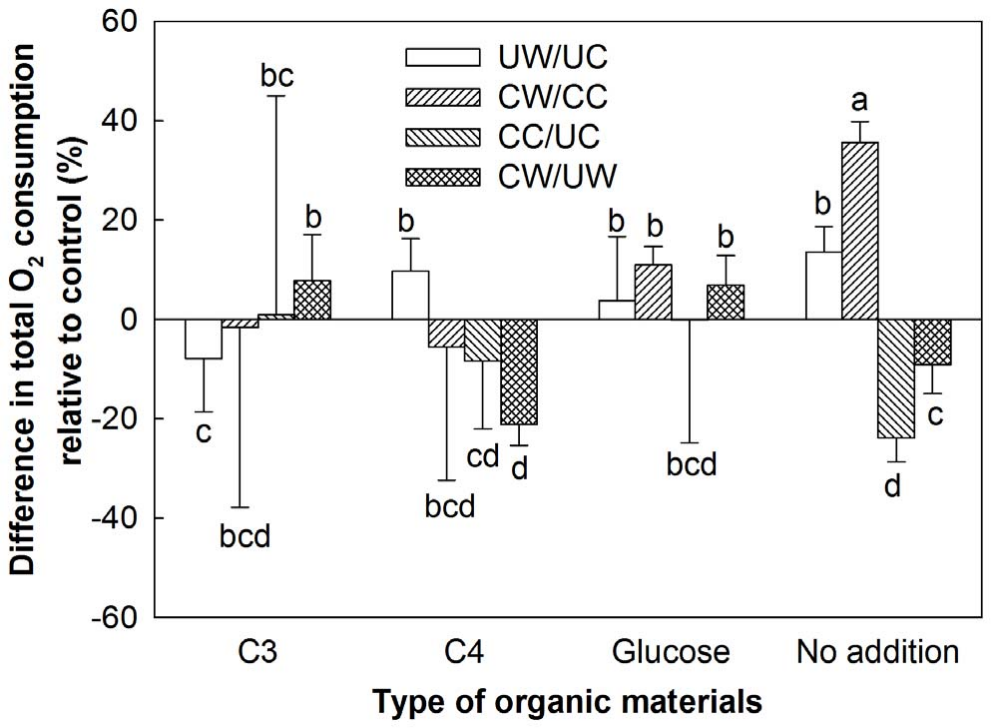
Warming- and clipping-induced changes in soil respiratory C release (%) with the addition of C_3_ plant material, C_4_ plant material, or glucose or without substrate addition during the incubation period under four treatments: UC: unclipped control; UW: unclipped and warmed; CC: clipped and control; CW: clipped and warmed. The comparisons included the effects of warming on soil respiratory C release with (CW/CC) and without (UW/UC) clipping, and the effects of clipping under control (CC/UC) and warming (CW/UW) treatments. Vertical bars and their error bars represent means and standard errors (*n* = 4). The different letters indicate statistical significance.

In soils without substrate addition, warming significantly increased soil respiratory C release and clipping decreased it over the whole incubation period (p<0.05), although the respiration rates declined over time (Fig. 1a). The respiration rates in the CW plots were close to those in the UC plots at the initial incubation stages and to those in the UW plots at the later incubation stages. The difference in soil respiratory C release between the UW and UC plots was statistically significant (p<0.01), whereas that between CW and UW plots was not different (p>0.05, Figs. 2). Warming significantly increased soil respiratory C release by 13.6 and 35.6% in the unclipped (UW vs. UC) and clipped (CW vs. CC) plots, respectively (p<0.05, Fig. 2). Clipping decreased the respiratory rate by 23.89% (p<0.05, CC vs. UC) in the unwarmed treatment but the decrease was not significant in the warmed plots (9.16%, p = 0.10, CW vs. UW) over the incubation period (Fig. 3).

The addition of glucose remarkably increased soil respiratory C release in all four treatments, but the effluxes declined more rapidly compared to those without added substrates (Fig. 1). The respiration rates in the CW plots were significantly higher than those in the CC and UC plots during the entire incubation period (p<0.05, Fig. 2). With the addition of glucose, the effects of warming/clipping on CO_2_-C respired were positive in the first week, but the respiration rates of all treatments were very similar at the final incubation stages (Fig. 1b). As a result, warming increased the respiration rate by 11.02% with clipping (p<0.01, CW vs. CC) with added glucose, but did not affect it without clipping (3.76%, p = 0.45, UW vs. UC). Clipping increased the respiration rate by 6.90% (p<0.05, CW vs. UW) under warming but had no effect in the unwarmed plots (Figs. 2, 3, CC vs. UC).

When the C_3_ materials were added, warming exhibited a positive effect on the respiration rates within the first week but a negative one thereafter (Fig. 1c). Both warming and clipping did not significantly affect total soil respiratory C release during the entire incubation period (p>0.05, Fig. 2). However, the respiration rates from the clipped soils were lower than those from the unclipped soils during the first week but they had the opposite response after one week, irrespective of the warming treatment (Fig.1c).

Following addition of the C_4_ plant materials, warming increased the respiration rate by 9.7% without clipping (p<0.05, UW vs. UC) but did not affect it with clipping (5.55%, p = 0.52, CW vs. CC, Fig. 1d). Clipping decreased the respiration rate by 21.1% under warming throughout the whole incubation period with the addition of C_4_ plant materials (p<0.05, CW vs. UW, Fig. 1d). The respiration rates in the clipped plots without warming were lower than those in the unclipped plots at the initial incubation stages but clipping increased it at the final incubation stages (Fig. 1d), resulting in the insignificant effects (8.3%, p = 0.72, CC vs. UC, Figs. 2, 3). Therefore, the respiration rates were highest in the UW plots and lowest in the CW plots (Fig. 2).

**Figure 4:**
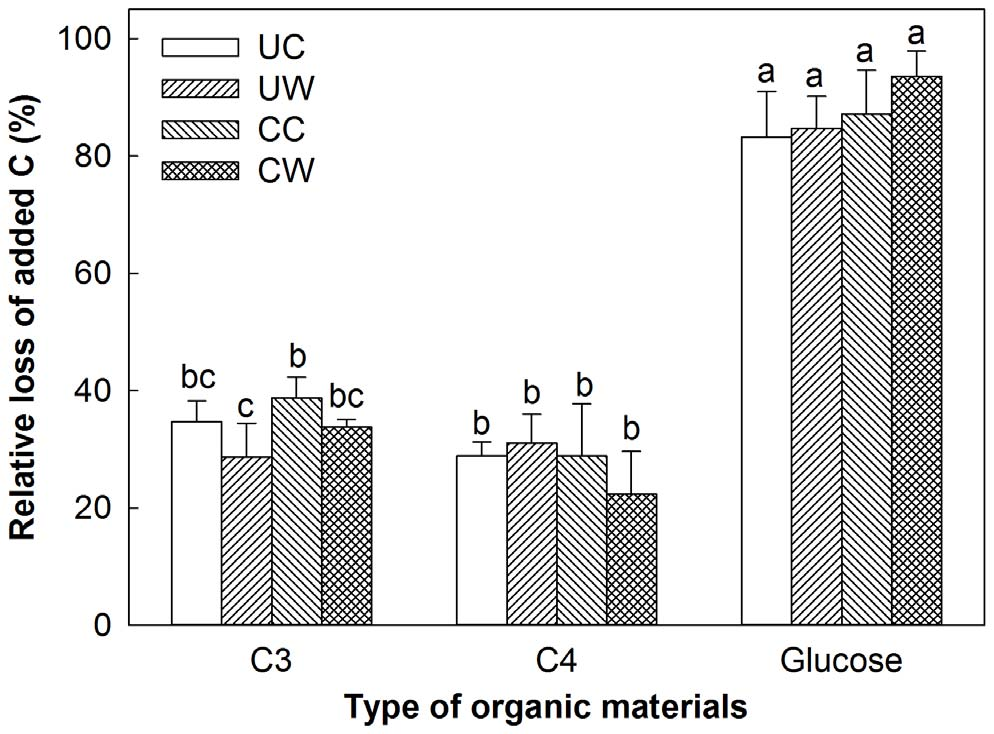
Warming- and clipping-induced relative loss of added C (i.e., glucose, C_3_ and C_4_ plants) compared to no additional substrate on soil respiratory C release (i.e., percent changes in soil respiratory C release between added C and no added substrate) under the four treatments. UC: unclipped control; UW: unclipped and warmed; CC: clipped and control; CW: clipped and warmed. Vertical bars and their error bars represent means and standard errors (*n* = 4). The different letters indicate statistical significance.

Compared to the control without substrate addition, C input to soils under three treatments (UC, CC and CW) significantly increased soil respiratory C release, whereas the relative loss of respiration induced by additional substrates differed significantly among the added materials (glucose> C_3_> C_4_ materials). In the UW plots, the relative loss of added C with additional glucose was the highest among the three additional substrates, whereas the loss from C_4_ materials was higher than that from C_3_ materials, although the quality of C_3_ material was higher than that of C_4_ material (Fig. 4). Warming decreased the decomposition of C_3_ plant materials but clipping increased it (Fig. 4). Warming only stimulated the decomposition of C_4_ plant materials without clipping but reduced it under clipping, although these effects were not significant.

## Discussion

### Effects of substrate addition on soil respiratory C release

The effects of warming and clipping on soil respiratory C release were largely regulated by the quality and quantity of substrates [15], [44]. Our results showed that warming increased soil respiratory C release (represented by O_2_ consumption) but clipping decreased it in the tallgrass prairie after 10 years of warming and clipping treatments, which was consistent with field measurements of heterotrophic respiration [20], [45]. The higher soil C release from the warmed plots compared to the controls in our study was attributable to differences in the soil C pools [17], [45]. The declined respiration rates over the incubation time were largely due to labile C depletion [20], [46].

Warming-induced increases in plant biomass growth, litter input, and root exudation may increase labile components in soil C pool [16], [17], [47], whereas clipping-induced reduction in aboveground NPP decreases labile C input to soil [17], [45]. In addition to the differences in labile C between the warmed and unwarmed plots, warming-induced increase in respired CO_2_ (Fig. 1a) may also be attributed to the decomposition of soil recalcitrant C. The respiration rates in the clipped and warmed (CW) plots was close to those in the unclipped control (UC) plots at the initial incubation stage, probably due to the effects of warming on soil labile C being offset by clipping [17]. Thereafter, the highest respiration rates were in the CW plots, largely owing to warming-induced increase in the decomposition of recalcitrant C pools [20]. The changes in the ability to decompose recalcitrant C under warming probably resulted from the long-term physiological adaption of microbes to changes in substrate supply [45]. Warming-induced dominance of C_4_ species in plant community caused a lowered quality of litter, inducing the corresponding adaptation in microbes [16]. In our experiment, when no substrate was added, clipping enhanced the effect of warming on soil respiratory C release (CW vs. clipped control [CC]) and warming weakened the effect of clipping without additional substrate (CW vs. unclipped and warmed [UW], Fig. 3), which was probably caused by the difference in soil C pools due to the positive interactions of warming and clipping on belowground NPP [16], [47].

Substrate inputs increased soil CO_2_ release, but the magnitudes of the effects varied largely with substrate types (glucose> C_3_> C_4_), probably resulting from the different quality of the added materials. On one hand, soil CO_2_ release following the substrate inputs was primarily controlled by the labile rather than total C pool [48]. Substrate input to the soil could induce high substrate availability, significantly causing higher respiratory C release than that seen in soils without additional substrate (Figs. 1, 2). The higher soil CO_2_ release under glucose addition was due to the fact that glucose is more readily decomposable than plant materials (C_3_ and C_4_). In contrast, C_4_ materials, with a higher C:N ratio than C_3_ materials, led to relatively low soil CO_2_ release. The quality of added substrates thus affected the decomposition rate of SOM and its palatability for soil microbes. On the other hand, soil microbial activity is commonly limited by nutrient availability and many soil organisms have very low metabolic rates, which spend most of their lifetime in dormant or resting phases [49], [50]. The respiratory C release could thus be significantly stimulated by adding easily degradable substrates due to stimulation in microbial metabolism. Changes in microbial community structure was not likely during the incubation, since the abundance of microbial genes did not change significantly with added substrates in our study (data not shown). However, changes in microbial composition can be a long-term regulatory mechanism, as demonstrated by our previous finding that long-term field treatments (warming and/or clipping) significantly affected the abundance of bacteria, fungi and archaea [39].

Warming-induced responses in soil CO_2_ release may be regulated by shifts in the plant community [16]. In the UW plots, total soil respiratory C release was relatively higher with additional C_4_ materials (5661 µg g^−1^) than with C_3_ materials (5448 µg g^−1^) with marginal significance (p = 0.09), even though C_3_ plant materials had a higher quality with a lower C:N ratio (Fig. 2). This suggest that, under long-term experimental warming, soil microbial communities have adapted to the dominant C source (C_4_ substrate) [51], [52]. Microbial community structure may be specifically selected, at least in part, by C inputs with more recalcitrant C_4_ materials in the past because warming stimulated C_4_ plant growth but depressed C_3_ plants [15]. Thus, warming depressed the decomposition of C_3_ litter in both clipped and unclipped plots (Fig. 4), resulting in low respiration rates (Fig. 3) compared with those in the unwarmed plots.

Land use practices such as mowing or clipping for hay in grassland may also have considerable effects on CO_2_ release in soils even under the addition of substrates with different quality. In our experimental site, soil microbial activity was restricted by a limited substrate supply due to low C and nitrogen (N) content (Table 1). In such oligotrophic soil environments, lack of available C is a primary constraint on soil microbial growth and activity [53], [54]. Clipping is likely to exacerbate the extent of C limitation for soil microbes and constrain microbial responses. Upon a sudden increase in C availability, microbes can apparently store a substantial amount of C in intracellular fluid and/or transfer C to cell structures (e.g., C addition, [55]). In our study, the added C_3_ and C_4_ plant materials may have been incorporated into structural components of microorganisms, leading to lower respiration rates in the clipped plots at the early incubation stage compared to the unclipped plots. As the substrate supply and microbial demand approached equilibrium, soil respiratory C release is likely to be dependent on the replenishment of labile substrates from the bulk organic C pool [56]. The higher respiration rates in the clipped plots compared to those in the unclipped plots at the later incubation stages were largely a consequence of compensatory growth and/or the physiological responses of microbes [57] at the early incubation stage. Here the compensatory point is defined as the point at which the negative effects of clipping on respiration rate becomes positive during the late stages of the incubation under added materials, which was determined by the degradable degree of the added materials. For example, the compensatory point occurred during the early incubation period at the end of the first week with the addition of glucose and four weeks after the start of incubation with the addition of C_3_ and C_4_ materials. The compensatory effects of clipping on the microbes were longer under warming than those under the controls with the same type of added materials (Figs. 1b, c, d). In the clipped plots, the increase in soil temperature and the decrease in soil labile C may alter the ability of the soil microbial community to utilize more recalcitrant C than labile C, especially under C-limiting conditions [6], [58], [59], resulting in more soil CO_2_ release during the later stage of incubation.

The addition of readily available substrates, such as glucose, significantly increased respiratory C release in all four treatments because glucose can be easily used by all microbial communities. Although the addition of native plant materials also increased respiratory C release, the shift of microbial composition indicated selective decomposition for substrates. These responses probably reflected different mechanisms or physiological strategies used by microbes to maintain their activity and vitality in response to changes in plant species. Climate warming associated with land management (e.g., clipping) may have potential to alter patterns of substrate utilization. For example, soil microbial community under the warming treatment had a higher ability to decompose C_4_ materials but, under the warming and clipping treatment (CW), it reduced this kind of capacity compared with the control (Fig. 4). Long-term warming may decrease the ability of microbes to use C_3_ plant materials relative to C_4_ ones, whereas clipping probably increases this ability with an insignificant trend (Fig. 4). This probably is due to warming- and/or clipping-induced variations in the microbial affinity for different substrates. Warming-induced changes in microbial community structure have the ability to metabolize substrates that are not used by members of the microbial community at lower temperature, and soil C release could thus potentially increase. The adaptation of microbes to higher temperature conditions may gradually adjust their metabolism. Adaptation of the soil microbial community to past recalcitrant C_4_ materials [35] cannot be ruled out as a mechanism for explaining the response of soil respiratory C release to warming.

### Implications for climate–C cycle feedback

The changes in substrate and microbial metabolic capacity under long-term warming and clipping could have profound impacts on grassland C balance. Without any additional substrate input, the significant effects of experimental manipulation in the field on incubated respiration rates confirmed the importance of factors other than temperature (e.g., substrates). The feedback of C cycles to warming are regulated by shifts of substrate supply and microbial community composition, which may play critical roles in soil C decomposition and become one of the major uncertainties in projecting future climate change [16]. Concurrent increases in soil respiratory C release and plant production induced by warming may cause fewer changes in the amount of labile and recalcitrant C in the soil. The positive warming effect on SOM decomposition may be weakened by shifts in plant species composition and increased residence time of SOM due to lowered litter quality [24], [60]. However, in our study, warming increased respiratory C release with the addition of C_4_ plant materials (p<0.05, Fig. 2) but relatively decreased it with the addition of C_3_ materials (insignificant, p>0.05), although C_4_ plant materials had lower quality than C_3_ ones. Our results revealed that warming-enhanced decomposition of recalcitrant C may offset warming-induced decreases in litter quality. Thus, the thermal adaptation of microbial communities to recalcitrant C_4_ materials will gradually become an important factor in regulating soil respiratory C release.

Clipping directly reduces the substrate input to soil [45], causing decreases in respiratory C release. In the clipped plots, the high-quality C input (e.g., glucose) may have rapidly provided available C to microbes and may have stimulated more C turnover in microbial biomass. The substrate-induced C release was lower with added C_4_ materials than with added C_3_ materials compared to additional glucose under clipping, indicating that more C may be potentially preserved in the soil due to high recalcitrance of C_4_ plant materials. However, the effects of biofuel feedstock harvesting on soil respiratory C release may need to be carefully considered in comparison with those of the clipping treatment. If energy use efficiency of producing bioenergy decreases with C_4_ plant materials due to their low quality, this may have similar effects to C loss. Future studies should include this extended component to evaluate a more general C budget. Our results suggested that both the quantity and quality of plant C input through litter fall or root exudates and the soil microbial capacity for decomposing substrate should be considered in modeling for predicting global warming.

In the future, changes in temperature could lead to long-term changes in the quality and quantity of plant inputs to soil due to the shift of plant species composition under warming. Alteration of microbial metabolic capacity in association with this change may exert a longer-term regulation on SOC. In our study, soil respiratory C release during the incubation period was significantly higher with supplementary C_4_ substrate than with C_3_ one for soils from field warming treatments without clipping (p<0.05, Fig. 4). This is likely to be linked to the plant community shift toward more C_4_ species under warming, indicating that soil microbial communities had already adapted to a niche with more dominant C_4_ species in the field. The potential negative feedback mechanism between warming and the C cycle due to the low quality of C_4_ materials might be abated by this adaptation of microbial communities. Moreover, clipping exaggerated the stimulating effect of field warming on the respiration rate during incubation. This continuing influence of the field treatments, consistent with field measurements of CO_2_ flux, may be attributed to their accumulated effects on soil substrate and/or microbial metabolic capacity. Future global biogeochemical models need to incorporate the indirect effects induced by climate warming, especially changes in the substrate supply and the microbial community.
